# The neural and genetic underpinnings of different developmental trajectories of Attention-Deficit/Hyperactivity Symptoms in children and adolescents

**DOI:** 10.1186/s12916-024-03449-1

**Published:** 2024-06-03

**Authors:** Yanpei Wang, Leilei Ma, Jiali Wang, Yuyin Ding, Ningyu Liu, Weiwei Men, Shuping Tan, Jia-Hong Gao, Shaozheng Qin, Yong He, Qi Dong, Sha Tao

**Affiliations:** 1 State Key Laboratory of Cognitive Neuroscience and Learning, Beijing Normal University, Beijing, 100875 China; 2 IDG/McGovern Institute for Brain Research, Beijing Normal University, Beijing, 100875 China; 3 Center for MRI Research, Academy for Advanced Interdisciplinary Studies, Peking University, Beijing, 100871 China; 4 Psychiatry Research Center, Beijing HuiLongGuan Hospital, Peking University, Beijing, 100096 China

**Keywords:** ADHD, Symptom trajectory, Neurodevelopmental, Genetic, Children and adolescents

## Abstract

**Background:**

The trajectory of attention-deficit hyperactivity disorder (ADHD) symptoms in children and adolescents, encompassing descending, stable, and ascending patterns, delineates their ADHD status as remission, persistence or late onset. However, the neural and genetic underpinnings governing the trajectory of ADHD remain inadequately elucidated.

**Methods:**

In this study, we employed neuroimaging techniques, behavioral assessments, and genetic analyses on a cohort of 487 children aged 6–15 from the Children School Functions and Brain Development project at baseline and two follow-up tests for 1 year each (interval 1: 1.14 ± 0.32 years; interval 2: 1.14 ± 0.30 years). We applied a Latent class mixed model (LCMM) to identify the developmental trajectory of ADHD symptoms in children and adolescents, while investigating the neural correlates through gray matter volume (GMV) analysis and exploring the genetic underpinnings using polygenic risk scores (PRS).

**Results:**

This study identified three distinct trajectories (ascending-high, stable-low, and descending-medium) of ADHD symptoms from childhood through adolescence. Utilizing the linear mixed-effects (LME) model, we discovered that attention hub regions served as the neural basis for these three developmental trajectories. These regions encompassed the left anterior cingulate cortex/medial prefrontal cortex (ACC/mPFC), responsible for inhibitory control; the right inferior parietal lobule (IPL), which facilitated conscious focus on exogenous stimuli; and the bilateral middle frontal gyrus/precentral gyrus (MFG/PCG), accountable for regulating both dorsal and ventral attention networks while playing a crucial role in flexible modulation of endogenous and extrinsic attention. Furthermore, our findings revealed that individuals in the ascending-high group exhibited the highest PRS for ADHD, followed by those in the descending-medium group, with individuals in the stable-low group displaying the lowest PRS. Notably, both ascending-high and descending-medium groups had significantly higher PRS compared to the stable-low group.

**Conclusions:**

The developmental trajectory of ADHD symptoms in the general population throughout childhood and adolescence can be reliably classified into ascending-high, stable-low, and descending-medium groups. The bilateral MFG/PCG, left ACC/mPFC, and right IPL may serve as crucial brain regions involved in attention processing, potentially determining these trajectories. Furthermore, the ascending-high pattern of ADHD symptoms exhibited the highest PRS for ADHD.

**Supplementary Information:**

The online version contains supplementary material available at 10.1186/s12916-024-03449-1.

## Background

Attention-deficit hyperactivity disorder (ADHD) is a neurodevelopmental disorder that typically emerges in childhood [1–3], and approximately 15% of individuals with an early diagnosis continue to meet clinical criteria for ADHD in adulthood [4]. While the prevailing understanding of ADHD focuses on two patterns of symptom persistence and remission, emerging research suggests that not all cases of adult ADHD are simply continuations from childhood [5–7]. This indicates the existence of another developmental pattern known as late-onset ADHD, which does not fulfill diagnostic criteria during childhood and adolescence but exhibits persistent ADHD symptom scores until diagnosis occurs in early adulthood [8]. Previous studies investigating the developmental trajectory of ADHD symptoms have commonly identified three trajectories: ascending, stable, and descending [9, 10]. However, these studies did not consider the potential influence of emotional and behavioral issues on developmental trajectories or explore underlying neural and genetic mechanisms. Therefore, this study aims to investigate both the stability of trajectory classification and elucidate the neurobiological and genetic foundations that underlie the developmental course of ADHD symptoms.

The development of the human brain entails significant alterations, particularly in the emergence and maturation of highly interconnected hub regions that facilitate integrated brain function [11]. These regions undergo an extended period of development extending into adulthood, during which abnormalities in the hub brain region can manifest as symptoms of ADHD. For example, one study reported that children and adolescents with ADHD exhibited lower cortical degree and betweenness in both the default mode network (DMN) and ventral attention network (VAN) compared to healthy controls [12]. Even in adulthood, weakened hub brain regions persist as a prominent functional characteristic among individuals with ADHD [13], including adults who had childhood-onset ADHD [14]. This suggests that changes occurring within hub brain regions may serve as crucial biomarkers influencing the developmental trajectory of ADHD characteristics. Therefore, we propose that key brain regions involved in attention processing may underlie the dynamic changes observed in ADHD characteristics.

The existing literature has demonstrated that ADHD is characterized by various functional and structural neural network abnormalities, particularly in the fronto-striatal circuitry [15–22]. Within the frontal cortex, both the middle frontal gyrus (MFG) and inferior frontal gyrus (IFG) have been identified as crucial hubs connecting the dorsal attention network (DAN) and VAN [23]. Specifically, the MFG plays a pivotal role in regulating both networks and flexibly modulating endogenous and extrinsic attention [24]. Improved remission of ADHD symptoms has been associated with enhanced node efficiency within the right MFG [25]. Moreover, neuro-functional activation patterns in the right MFG hold promise as potential biomarkers for monitoring acute effects of methylphenidate treatment in children with ADHD [26]. A series of meta-analyses and mega-analyses based on structural or functional imaging collectively demonstrate that individuals with ADHD exhibit smaller striatal volumes and reduced activation compared to healthy controls [18, 19, 22, 27–33]. In a comprehensive study incorporating both structural and functional image meta-analysis, patients with ADHD showed decreased GMV and diminished activation specifically in the right putamen when compared to controls [34]. The dysfunction within the prefrontal-striatal circuitry encompassing the striatum and prefrontal lobe has long been recognized as a fundamental neuropsychological basis for ADHD [35, 36]. ADHD is also believed to be associated with the DMN of activities that require effortful engagement [37]. Several studies have identified dysregulation of DMN as a crucial neurobiological basis for recognizing deficits in individuals with ADHD [38, 39]. Some scholars argue that ADHD can be considered as a disorder of DMN [40], which has been supported by a mega-analysis of multiple large samples [41]. They hypothesize that the DMN in individuals with ADHD may lack regulation from other neural systems, leading to disruptions in ongoing cognition and behavior, resulting in periodic lapses in task performance—a hallmark feature of ADHD [42]. Furthermore, abnormal activation of the dorso-ventral attention network during response inhibition [43, 44], reduced volume of the visual cortex [45], and weakened motor network activation [46] have also been observed as significant deficits in individuals with ADHD compared to healthy controls. We speculate that these differences may arise due to alterations within hub nodes across these networks contributing to distinct developmental trajectories of ADHD symptoms.

Previous studies have provided compelling evidence supporting the genetic basis of ADHD, with heritability estimates ranging from 0.76 to as high as 0.9, which is the highest among psychiatric disorders [47, 48]. Twin studies have further substantiated the heritability of ADHD symptoms, and advancements in genomic research now enable direct assessment of genetic contributions [49–52]. Genomic investigations have identified multiple common risk alleles and rare mutations that contribute to the underlying genetic architecture of ADHD [53]. Although individual common risk alleles typically exhibit modest effect sizes in multifactorial disorders like ADHD, composite measures such as PRS offer valuable biological indicators by estimating an individual's cumulative burden of common risk alleles based on their association statistics and effect sizes derived from genome-wide association studies [52]. PRS for ADHD demonstrate higher values in patients with the disorder compared to controls [54] and are associated with levels of ADHD symptoms within the general population [55, 56]. A study investigating the developmental trajectories of ADHD has revealed that the persistence of ADHD symptoms throughout childhood and adolescence in the general population is associated with a higher PRS for ADHD [57]. However, by dichotomizing symptom scores into binary data to determine the presence or absence of ADHD, valuable graded information is lost, impeding the identification of individuals with heightened symptoms and hindering early prevention and intervention efforts for those at high risk but without a confirmed diagnosis of ADHD. In this study, we utilized symptom scores to examine whether different developmental trajectories of ADHD symptoms throughout childhood and adolescence in the general population are influenced by PRS for ADHD.

Therefore, the aims of this study were 1) to identify and classify distinct developmental trajectories of ADHD symptoms using longitudinal data spanning 3 years; 2) to investigate the neural mechanisms underlying these developmental classifications through intergroup comparisons; and 3) to examine the potential contribution of PRS for ADHD to different developmental trajectories. To accomplish these aims, we utilized a longitudinal cohort comprising children and adolescents who underwent three-wave imaging and behavioral assessments, along with genetic data. We hypothesized that ADHD symptoms would exhibit diverse developmental trajectories determined by hub brain regions, which could also be influenced by PRS for ADHD.

## Methods

### Design and sample

Neuroimaging and behavioral data were obtained from the Children School Functions and Brain Development project (CBD, Beijing Cohort), which is a longitudinal cohort [58]. Baseline data used in the present cohort study were acquired from July 28, 2016, to October 26, 2019, first follow-up data were acquired from August 9, 2017, to April 11, 2021, and second follow-up data were acquired from July 27, 2018, to May 1, 2022. All children’s parents/guardians signed an informed consent form approved by the Ethics Committee of Beijing Normal University.

The participants aged 6–16 years underwent three-wave imaging and behavioral assessment. The two follow-up assessments were at 1 year (1-year follow-up) and 2 years (2-year follow-up) after the baseline assessment. We identified 487 participants who had behavioral and neuroimaging data available at the study baseline, 1-year follow-up, and 2-year follow-up, and all three brain images of 460 children passed quality control. All participants were recruited from primary schools in Beijing with normal cognitive ability, assessed by a well-validated Chinese standardized cognitive ability test [59]. Exclusion criteria included notable physical illness or head trauma. The pediatric expert system training staff used the Mini-International Neuropsychiatric Interview [60] to measure children’s mental state, excluding the medical conditions (including ADHD). The children were prohibited from taking any drugs or ingesting caffeine on the day of the behavioral tests and MRI scans.

### Clinical assessment

The parent-reported version of the Strengths and Difficulties Questionnaire (SDQ) was used to assess symptoms of hyperactivity and inattention[61]. The Chinese version was retrieved from the SDQ website (https://www.sdqinfo.org/py/sdqinfo/b3.py?language=Chinese). The SDQ is a reliable and valid tool for mental health problems in children and adolescents [61] and has been demonstrated in IMAGEN to be a promising assessment for ADHD symptoms [62–65]. The hyperactivity-inattention subscale is composed of five items covering three key symptom domains for ADHD; the subscale’s internal consistency (Cronbach’s alpha = 0.75) is at an acceptable level [66]. As used in nationwide epidemiological studies [67], a three-band classification was established for the SDQ using a cut-off score of 6 (normal: scores < 6; borderline: score of 6; abnormal: scores > 6). We used the parent-report SDQ because it is more reliable than the child self-report version, and the parent-report SDQ also has a stronger association with clinical assessments [61, 67].

### Structural MRI

The MRI acquisition protocols and quality controls in CBD have been described in detail [68]. A high-resolution T1-weighted magnetization-prepared gradient echo sequence was collected using 3-T scanners and preprocessed using the Computational Anatomy Toolbox (CAT12) (http://dbm.neuro.uni-jena.de/cat) and SPM12 (http://www.fil.ion.ucl.ac.uk/spm), as reported previously [68] (detailed MRI acquisition, quality controls, and image processing in Additional file 1: Method S1) [69].

### Genetic data

Genotyping was carried out from blood drawn from CBD participants. The blood collection and genetic data acquisition were successfully completed by a total of 1424 children. All individuals were genotyped on Infinium™Omni2.5–8 v1.5 BeadChip. After quality control, 1282 cases were included in our sample, totaling 69,842 single-nucleotide polymorphisms available for establishing the polygenic risk score (PRS) for ADHD. In this study, 473 children completed blood collection. Quality control of genetic data was in Additional file 1: Method S2.

### Statistical analyses

#### Latent class mixed model to detect ADHD trajectories

We used a latent class mixed model (LCMM) on ADHD symptoms data to ascertain different trajectories of ADHD symptoms from 487 individuals of the CBD cohort. LCMM was designed to differentiate of individuals following different developmental trajectories, providing specific trajectories, the number of individuals belonging to each trajectory, and the individual’s probability of belonging to specific trajectories. Since the maximum number of known groups of ADHD symptoms trajectories was 6 [70], therefore, the best-fitting solution between one class and seven classes was defined through the lowest Bayesian information criterion (BIC), and theoretical and clinical utility of the model. LCMM analyses were conducted with the R package lcmm (version 2.0.1) for the R software for Windows, version 4.3.1 [71]. The model syntax was in Additional file 1: Method S3.

#### Imaging statistical analysis

The GMV analysis was implemented using DPABI, a toolbox for MATLAB [72], and R 4.3.1. To quantify the effects of groups of ADHD symptoms and age on the GMV, we used a linear mixed model [73], which can characterize the main effects of groups of ADHD developmental trajectories and age, as well as the interaction between them. The model syntax was in Additional file 1: Method S3. A significance threshold set at a voxel-wise value of *p* < 0.001 and a family-wise error (FWE) corrected cluster probability of *p* < 0.05 were used for multiple comparison correction [74]. Sex, handedness, parental education, family income, IQR (image and preprocessing quality), and site were considered as covariates. IQ is not recommended as a variable to be controlled in cognitive studies of neurodevelopmental disorders, since it is often affected by the disorder [75].

#### Polygenic analysis

The latest genome-wide association meta-analysis of 38,691 patients with ADHD and 186,843 control subjects was used as the discovery data set [76]; the summary statistics were downloaded from the Psychiatric Genomics Consortium (https://pgc.unc.edu/for-researchers/download-results/). Polygenic risk score analyses were performed using PRSice-2 after extracting 10 principal components along with age and gender as covariates [77]. PRS association *p* values were corrected for multiple testing using the Benjamini and Hochberg false discovery rate (FDR) method as implemented in the R framework stats package [78]. When comparing differences in PRS for ADHD between groups with distinct trajectories of symptom development, a *p*-value ranging from 0.05 to 0.10 was considered marginally significant [79].

## Results

### Demographics and LCMM trajectories of ADHD symptoms

Demographic information is summarized in Table 1. Relative to baseline, hyperactivity-inattention total score was gradually reduced for both 1-year and 2-year follow-ups, as did the proportion of abnormal scores.

**Table 1:** Summary statistics for demographic variables

		Baseline (*n* = 487)	1-year follow-up (*n* = 487)	2-year follow-up (*n* = 487)
Age, mean (SD)		9.16 (1.43)	10.30 (1.51)	11.44 (1.58)
Sex, females, *n* (%)		209 (42.92%)		
Parental education, mean (SD)^a^		5.81 (1.34)		
Family income, mean (SD)^b^		7.63 (1.66)		
Cognitive ability, mean (SD)		95.61 (12.17)	101.80 (12.64)	108.49 (12.21)
Hyperactivity-inattention total score, mean (SD)		4.15 (2.48)	3.72 (2.21)	3.37 (2.18)
ADHD categories by hyperactivity-in attention total score^c^,* n* (%)	Normal	352 (72.28%)	377 (77.41%)	412 (84.60%)
	Borderline	51 (10.47%)	52 (10.68%)	32 (6.57%)
	Abnormal	84 (17.25%)	58 (11.91%)	43 (8.83%)

^a,b^Details for the parental education and family income score can be found in Additional file 1: Method S4

^c^Scores were categorized as follows: normal: score < 6; borderline: score = 6; abnormal: score > 6

**Fig. 1:**
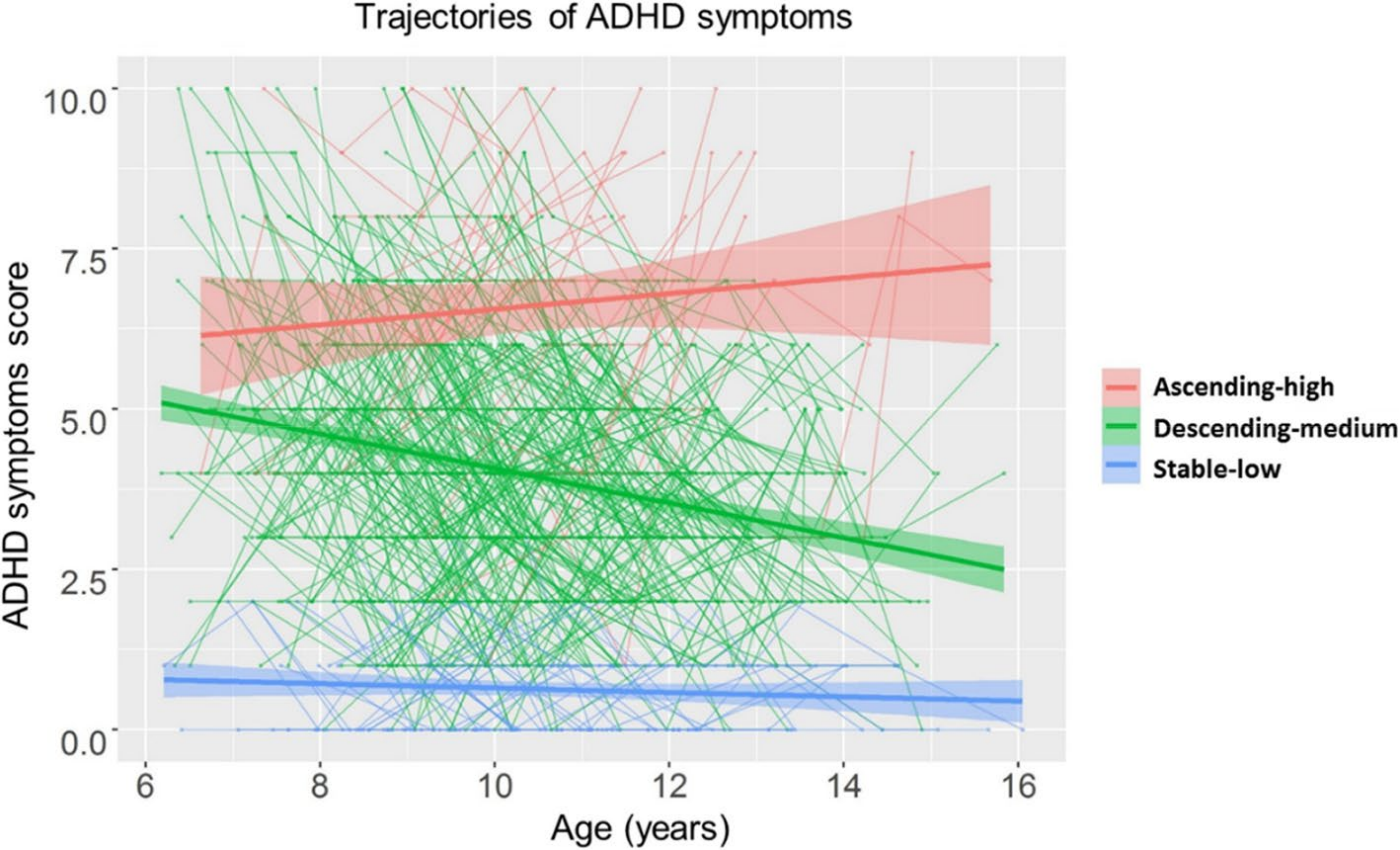
The different developmental trajectories of ADHD symptoms. The shaded areas represent the 95% confidence intervals. Individual participants are represented by individual lines, and participants measured once are represented by dots

When taking into account the entire cohort, the best model comprised three trajectories: 7.60% (ascending), 13.35% (stable), and 79.06% (descending) (Additional file 1: Table S1). In addition to the difference in trajectories, we also examined the difference in the absolute value of the three trajectories and found that there were significant differences among the three groups (*F*_(2,1458)_ = 425.29, *η*^2^ = 0.37, *p* < 0.001). The post hoc test analysis showed that the ascending group (mean ± SD: 6.60 ± 2.09) > the descending group (mean ± SD: 4.00 ± 1.94) > the stable group (mean ± SD: 0.63 ± 0.77). Therefore, we defined the three groups as: ascending-high, descending-medium, and stable-low (Fig. 1). Then, we used a LME model to fit the age effects under the three trajectories and found that ADHD symptoms increased significantly with age in the ascending-high group (*β* = 0.38, *p* < 0.001), no age effect in the stable-low group (*β* =  − 0.10, *p* = 0.181), and ADHD symptoms decreased significantly with age in the descending-medium group (*β* =  − 0.32, *p* < 0.001). The detailed information of ascending-high, stable-low, and descending-medium is summarized in Table 2. Age has no main effect on the trajectory group (*F*_(2,968)_ = 1.55,* η*^2^ < 0.01, *p* = 0.214) and no interaction effect between the trajectory group and the time point (*F*_(4,968)_ = 1.56, *η*^2^ < 0.01, *p* = 0.183). The parental education showed a significant difference in the trajectory group (*F*_(2,484)_ = 5.33, *η*^2^ = 0.02, *p* = 0.005), and the parental education in the ascending-high group was significantly lower than that in the stable-low group (mean difference = 0.87, *p* = 0.002). Family income showed no difference in the trajectory group (*F*_(2,484)_ = 0.10,* η*^2^ < 0.01, *p* = 0.908). Cognitive ability has a significant main effect on the trajectory group (*F*_(2,968)_ = 222.03,* η*^2^ = 0.37, *p* < 0.001) and no interaction effect between the trajectory group and the time point (*F*_(4,968)_ = 2.15, *η*^2^ = 0.01, *p* = 0.073). The post hoc test analysis showed that the stable group > the descending group > the ascending group in the cognitive ability.

**Table 2:** Demographic variables of ascending-high, stable-low, and descending-medium group

		Baseline (*n* = 487)	1-year follow-up (*n* = 487)	2-year follow-up (*n* = 487)
Age, mean (SD)	Ascending-high	9.08 (1.17)	10.15 (1.28)	11.24 (1.27)
	Stable-low	9.43 (1.58)	10.59 (1.65)	11.75 (1.73)
	Descending-medium	9.12 (1.43)	10.26 (1.50)	11.41 (1.58)
Sex, females, *n* (%)	Ascending-high	12 (32.43%)		
	Stable-low	28 (43.08%)		
	Descending-medium	169 (43.90%)		
Parental education, mean (SD)^a^	Ascending-high	5.35 (1.39)		
	Stable-low	6.22 (1.15)		
	Descending-medium	5.78 (1.35)		
Family income, mean (SD)^b^	Ascending-high	7.51 (1.79)		
	Stable-low	7.63 (1.74)		
	Descending-medium	7.64 (1.64)		
Cognitive ability, mean (SD)	Ascending-high	90.71 (12.09)	96.82 (12.81)	100.02 (13.36)
	Stable-low	102.61 (11.7)	110.74 (10.5)	115.72 (11.62)
	Descending-medium	94.9 (11.83)	100.83 (12.32)	108.03 (11.59)
Hyperactivity-inattention total score, mean (SD)	Ascending-high	5.73 (2.28)	6.41 (1.98)	7.68 (1.51)
	Stable-low	0.85 (0.87)	0.60 (0.79)	0.43 (0.59)
	Descending-medium	4.55 (2.21)	3.99 (1.81)	3.45 (1.58)
Emotional symptoms score, mean (SD)	Ascending-high	1.49 (1.88)	1.41 (1.61)	1.62 (1.66)
	Stable-low	0.80 (0.96)	0.63 (1.08)	0.54 (1.00)
	Descending-medium	1.54 (1.42)	1.18 (1.25)	0.92 (1.07)
Conduct problems score, mean (SD)	Ascending-high	2.05 (1.54)	2.16 (1.30)	2.54 (1.61)
	Stable-low	1.08 (0.91)	0.92 (0.78)	0.88 (0.86)
	Descending-medium	1.72 (1.29)	1.52 (1.12)	1.46 (0.99)

^a,b^Details for the parental education and family income score can be found in Additional file 1: Method S3

To determine the impact of emotional symptoms and conduct problems on the developmental trajectories of ADHD symptoms, we assessed the developmental trajectories of emotional symptoms and conduct problems by the ADHD symptoms group and found that they showed similar trajectories to ADHD symptoms (Additional file 1: Figure S1). In order to exclude the influence of emotional symptoms and conduct problems on the ADHD trajectory, we observed the three developmental trajectory subgroups of ADHD after controlling them as covariates and found that the outcomes were not affected (ascending-high:* β* = 0.38, *p* < 0.001; stable-low: *β* =  − 0.09, *p* = 0.229; descending-medium:* β* =  − 0.25, *p* < 0.001).

### The different developmental changes of GMV among descending-medium, stable-low, and ascending-high group

**Fig. 2:**
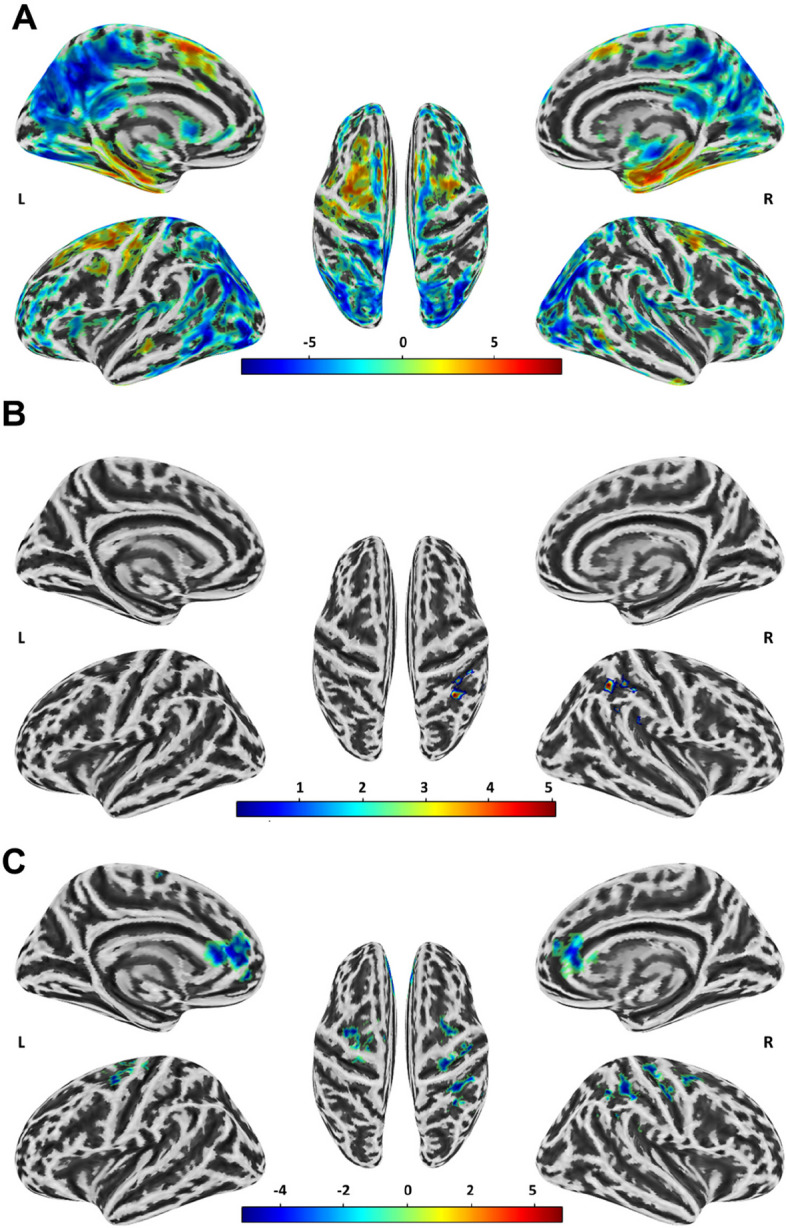
The different developmental changes of GMV among descending-medium, stable-low, and ascending-high groups. **A** The age effects on GMV. **B** The group (descending-medium, stable-low, and ascending-high) effect on GMV. **C** The interaction of age and group on GMV. GMV, gray matter volume. The color bar represents *z* value. L, left; R, right

**Fig. 3:**
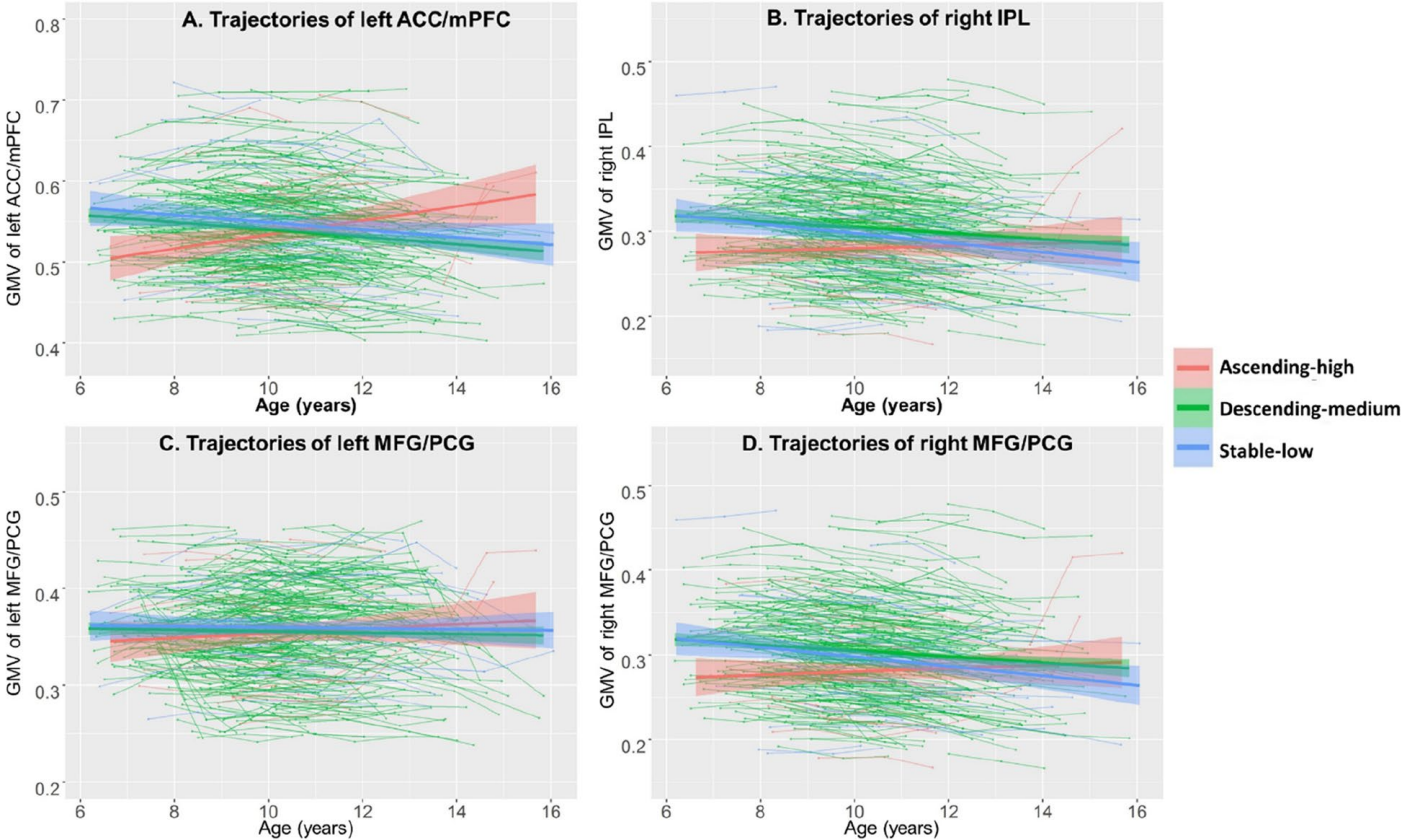
The different developmental trajectories of GMV in four clusters across descending-medium, stable-low, and ascending-high groups. **A** The developmental trajectory of GMV in left ACC/mPFC. **B** The developmental trajectory of GMV in the right IPL. **C** The developmental trajectory of GMV in left MFG/PCG. **D** The developmental trajectory of GMV in right MFG/PCG. GMV, gray matter volume; ACC/mPFC, anterior cingulate/medial frontal cortex; IPL, inferior parietal lobule; MFG/PCG, middle frontal gyrus/precentral gyrus. The shaded areas represent the 95% confidence intervals. Individual participants are represented by individual lines, and participants measured once are represented by dots

We use a LME model to explore the main effects of age, group, and their interaction on GMV. The results showed that age was significantly positively correlated with four clusters and negatively correlated with four clusters (Fig. 2A and Additional file 1: Table S2). A significant group effect was found in the right inferior parietal lobule (IPL, cluster size = 237, peak (MNI): 33, − 42, 39), Fig. 2B and Additional file 1: Table S3), and post hoc analysis found that the ascending-high group (0.291 ± 0.060) was significantly smaller than the stable-low group (0.317 ± 0.077, *p* = 0.0011) and the descending-medium group (0.331 ± 0.067, *p* < 0.001), and the stable-low group was significantly smaller than the descending-medium group (*p* = 0.006). The interaction effect between age and group was shown in four clusters: left anterior cingulate/medial frontal cortex (ACC/mPFC, cluster size = 485, peak (MNI): − 15, 48, 0), right IPL (cluster size = 338, peak (MNI): − 30, − 42, 39), right middle frontal gyrus/ precentral gyrus (MFG/PCG, cluster size = 362, peak (MNI): 33, − 21, 42), left MFG/PCG (cluster size = 255, peak (MNI): − 24, − 21, 66) (Fig. 2C and Additional file 1: Table S4). Then, we conducted an analysis on the developmental trajectories of the four clusters within the descending-medium, stable-low, and ascending-high groups. Our findings revealed a significant age-related decrease in GMV within the four brain regions in the descending-medium group (left ACC: *r* =  − 0.09, *p* < 0.001; right IPL: *r* =  − 0.14, *p* < 0.001; left MFG/PCG: *r* =  − 0.08, *p* < 0.001; right MFG/PCG: *r* =  − 0.10, *p* < 0.001). In contrast, the GMV within these regions either significantly decreased or remained unchanged with age in the stable-low group (left ACC: *r* =  − 0.05, *p* = 0.022; right IPL: *r* =  − 0.08, *p* < 0.001; left MFG/PCG: *r* =  − 0.02, *p* = 0.479; right MFG/PCG: *r* =  − 0.03, *p* = 0.132). Interestingly, in the ascending-high group, there was an observed increase or no change in GMV with age (left ACC: *r* = 0.07, *p* = 0.182; right IPL: *r* =  − 0.06, *p* = 0.362; left MFG/PCG: *r* = 0.13, *p* = 0.009; right MFG/PCG: *r* = 0.04, *p* = 0.351) (Fig. 3A–D).

### Contributions of PRS for ADHD

**Fig. 4:**
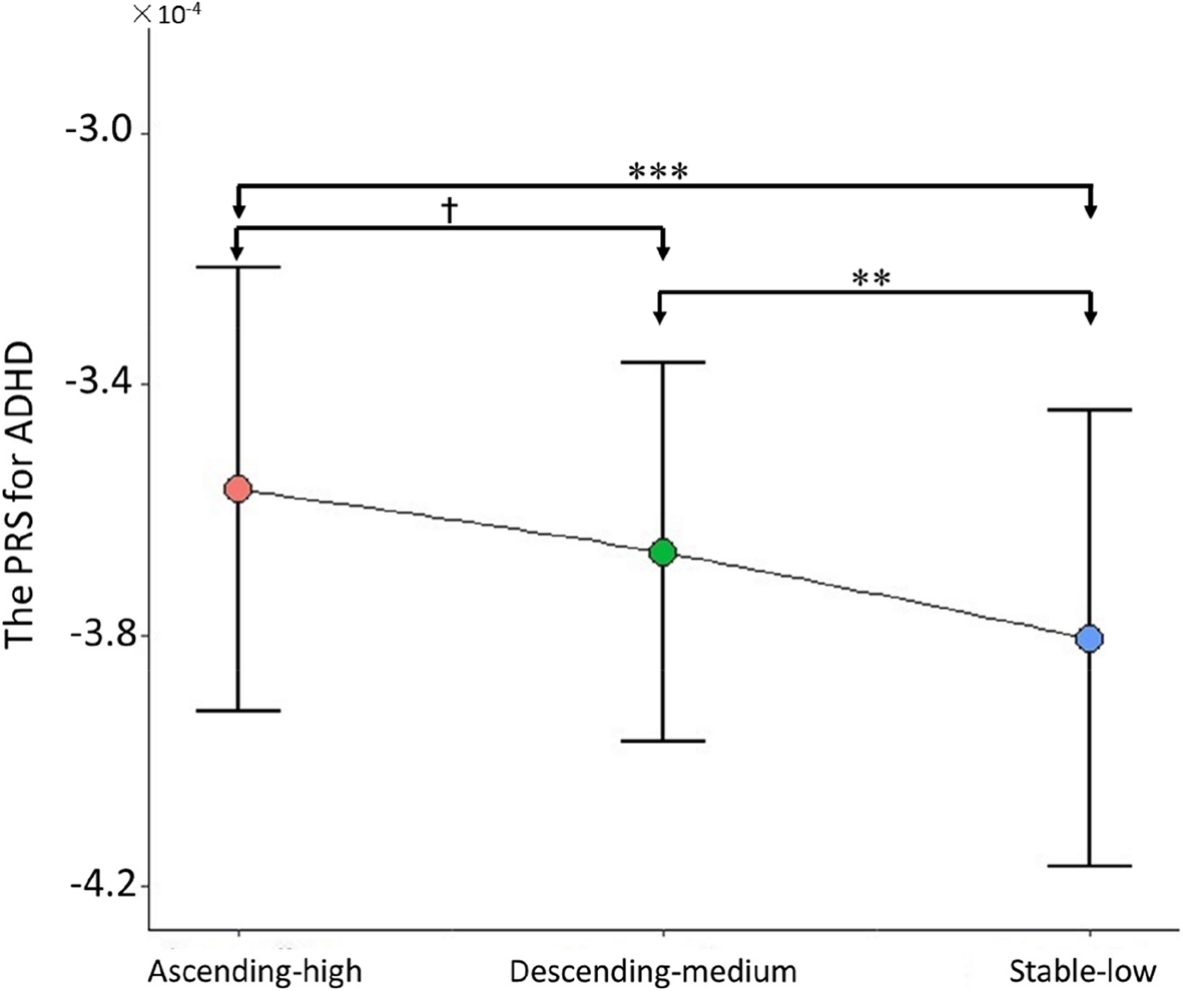
Mean PRS for ADHD by descending-medium, stable-low, and ascending-high group. Significant group comparisons are indicated with asterisks ✝0.05 < *p* < 0.10 (marginally significant); *p < 0.05; **p < 0.01; ***p < 0.001, PRS, polygenic risk score

In the genetic data, we found that the mean PRS for ADHD differed across the descending-medium (− 3.67 ± 0.30 × 10^−4^), stable-low (− 3.81 ± 0.37 × 10^−4^), and ascending-high group (− 3.57 ± 0.36 × 10^−4^) (*F*_(2,468)_ = 7.66, *η*^2^ = 0.03, *p* < 0.001, Fig. 4). Then the post hoc test analysis found pairwise differences in the mean PRS for ADHD, the PRS for ADHD in both the ascending-high (mean difference = 2.38 × 10^−5^, *p* < 0.001) and descending-medium group (mean difference = 1.37 × 10^−5^, *p* = 0.0014) were significantly higher than those in the stable-low group, while the difference between the ascending-high group and the descending-medium group reached marginally significant (mean difference = 1.01 × 10^−5^, *p* = 0.065).

## Discussion

Based on a 3-year longitudinal cohort dataset, we employed the LCMM to classify the developmental trajectory of ADHD symptoms in children and adolescents into three distinct groups: ascending-high, stable-low, and descending-medium. Utilizing the LME model, we identified attention hub regions such as the left ACC/mPFC, right IPL, and bilateral MFG/PCG that potentially determine these three groups with diverse developmental trajectories. Furthermore, significant differences were observed in PRS for ADHD across the ascending-high, stable-low, and descending-medium groups. These findings provide a neurobiological and genetic basis for understanding the distinct developmental trajectory of ADHD symptoms.

In this study, we successfully categorized the development of ADHD symptoms into three groups: ascending-high, stable-low, and descending-medium, which aligns with the findings reported by Breda et al. [9]. Although their study employed a larger sample size, the lack of consistent measurement tools to assess ADHD symptoms during follow-up raised concerns about result stability. To address this issue, our study utilized identical measurement tools and replicated their findings. The three groups identified in this study represent distinct populations that necessitate different treatment strategies. First, the stable-low group comprises children and adolescents exhibiting the fewest ADHD symptoms with long-term stability, indicating a favorable developmental trajectory without requiring attention. Second, the descending-medium group initially presented higher symptom scores but experienced further remission as neurodevelopmental maturation progressed, constituting the majority of participants. Of utmost concern is the ascending-high group, characterized by elevated ADHD symptoms at baseline and a subsequent increase over time. The findings from both the descending-medium and ascending-high groups emphasize the significance of continuous dynamic monitoring when encountering high symptom scores in children, and prompt intervention should be warranted if symptom scores are elevated to prevent these groups from developing late-onset ADHD.

Furthermore, previous research has indicated that emotional and conduct problems often influence the development of ADHD symptoms in children [80, 81]. We examined emotional and conduct problems among children in each group (ascending-high, stable-low, and descending-medium) of ADHD symptoms and observed a similar trajectory; however, this association was weaker compared to the developmental trajectory of ADHD itself. Importantly though when controlling for emotional and conduct problems' effects on symptom development, we found no impact on the developmental trajectories across all three groups. These results suggest that the identified groupings in our study are primarily driven by ADHD symptoms themselves rather than emotional or conduct problems.

Among the three distinct developmental trajectory groups, we observed significant differences in parental education levels, particularly with regards to the ascending-high group exhibiting significantly lower levels compared to the stable-low group. Both the meta-analysis and large sample study (*n* = 446,113) corroborated that low parental education was significantly associated with an elevated risk of ADHD [82, 83]. This finding aligns with our current study's observation that parents belonging to the ascending-high group had lower educational attainment and higher symptom scores. Furthermore, a study investigating developmental trajectories of ADHD symptoms revealed a primary association between parental education levels and ADHD symptom scores; specifically, individuals displaying higher symptom scores tended to have parents with lower educational backgrounds [84]. Notably, parental education level did not influence the trajectory of symptom development; for instance, those in the ascending-low group exhibited higher levels of parental education compared to individuals in the stable-high group. Our findings suggest that discrepancies in parental education primarily relate to variation in symptom severity rather than developmental trajectories.

The cognitive ability difference was observed among the three distinct developmental trajectories, namely, the ascending-high group exhibited the poorest cognitive ability, followed by the descending-medium group, while the stable-low group demonstrated superior cognitive ability. Previous studies have established that cognitive dysfunction is a significant impairment associated with ADHD [85] and that both ADHD and cognitive impairment share common genetic origins [86]. A population-based birth cohort study investigating different developmental trajectories of ADHD symptoms revealed an association between differences in cognitive ability and ADHD symptom scores; specifically, higher ADHD symptom scores at a given time point were correlated with poorer cognitive performance [87]. These findings align with our study's results, where we also identified differences among groups: individuals in the ascending-high group displayed the most severe ADHD symptoms along with the worst cognitive performance, whereas those in the stable-low group exhibited mildest ADHD symptoms alongside the best cognitive performance. This study further confirms that the relationship between cognitive ability and ADHD symptoms primarily depends on the severity of these symptoms rather than on their trajectory of change.

By analyzing the interaction effects of different developmental trajectory groups and age on GMV, we found four significant brain regions: the left ACC/mPFC, right IPL, and bilateral MFG/PCG. Further analysis revealed a significant age-related decrease in GMV within the four brain regions in the descending-medium group. In contrast, the GMV within these regions either significantly decreased or remained unchanged with age in the stable-low group. Interestingly, in the ascending-high group, there was an observed increase or no change in GMV with age. GMV reduction is usually explained by two theoretical models: pruning models [88] and myelination [89]. Pruning models propose that cortical atrophy reflects the loss or remodeling of synapses, dendrites, or cell bodies [88]. Myelination models suggest that the cortex seems to undergo shrinkage because of an increase in the proportion of myelinated axons and that the volume reduction does not necessarily mean any loss or change in neuronal material [89]. In this study, we found that the stable-low group with low symptom scores had a gradual decrease or no change in GMV, while the descending-medium group with high symptom scores but then a rapid decline showed a steady decline in GMV, suggesting that the decline in GMV represented by neuropruning and myelination is the brain basis for lower ADHD symptom scores or maintenance at lower levels. However, individuals belonging to the ascending-high group demonstrated either an increase or no change in GMV, suggesting potential impediments to neuropruning and myelination processes.

The ACC/mPFC serves as a central hub in the brain for integrating cognitive control and directing attention, affect, and motivation. Consequently, any anatomical alterations in this region may give rise to impulsivity, hyperactivity, and inattention—cardinal behavioral manifestations of ADHD. Notably, the GMV of the ACC was found to be significantly smaller (by 21% to 23%) in individuals with ADHD compared to healthy controls [90]. Furthermore, meta-analysis revealed that ACC function activation was significantly weaker than that observed in the healthy control group [19]. Additionally, given that the ACC is considered as one of the core brain regions within the default mode network (DMN), it is noteworthy that some scholars consider ADHD as a disorder affecting this network [40]. Reduced DMN consistency has indeed been identified in individuals with ADHD [91], while a small sample study on children with ADHD demonstrated an association between reduced DMN inhibition and increased intra-individual variability [92]. Therefore, abnormal development of the ACC/mPFC among individuals exhibiting heightened symptoms of ADHD can be viewed as indicative of impaired inhibitory control.

The MFG serves as the circuit breaker for the DAN and VAN, responsible for facilitating switching between these two networks [23, 24, 93, 94]. The coupling of DAN and VAN in healthy individuals has been shown to predict attention performance [95]. Abnormal development of the MFG/PCG, which acts as a central hub for switching between DAN and VAN, may result in inadequate cognitive resources necessary for efficient transitions between these networks. Disruptions in the coupling of DAN and VAN have been associated with attention deficits observed across various psychiatric disorders [96]. Furthermore, increasing the GMV of MFG through reading intervention has also been found to promote attentional development [68]. Therefore, the aberrant developmental pattern identified in the group exhibiting increased ADHD symptoms within this study may indicate impaired regulation of attention.

The IPL, as a core region of the temporo-parietal junction (TPJ), plays a crucial role in conscious-based attention control by manipulating consciousness to focus on stimulus targets and acting as top-down control over dorsal and ventral visual systems [97, 98]. Heightened activation in the right TPJ during attention processing exhibited a significant correlation with ADHD symptom scores [99]. Impairments in stimulus-driven orienting may arise from functional deficits of the TPJ [100]. The aberrant development of IPL observed in individuals with ascending-high ADHD symptoms within this study might indicate an impaired ability to shift attention towards unexpected stimuli or reorient attention.

As a crucial constituent of the fronto-striatal circuitry, previous meta-analyses have consistently reported decreased striatal volumes and weaker functional activation in individuals with ADHD compared to TD controls [18, 19, 22, 27–33]. However, this study exclusively identified abnormalities solely within the frontal cortex and did not observe the involvement of the striatum in the developmental trajectory of ADHD symptoms. We propose two potential explanations for this finding. First, our study employed a relatively stringent threshold, whereas adopting a more lenient threshold revealed involvement of the striatum. Second, it is well-established that striatal dysfunction plays a pathological role in ADHD, and a meta-analysis utilizing dopamine transporter imaging has confirmed the viability of targeting the striatum for medication [101]. Nevertheless, as our study examined the developmental trajectory of ADHD symptom scores in the general population, where most children may not exhibit abnormalities in dopamine transporters, any abnormalities in the striatum were not identified under the relatively stringent threshold used in this study.

In this study, we investigated the differences in PRS for ADHD among three subgroups characterized by distinct developmental trajectories of ADHD symptoms. Our findings revealed that the stable-low group exhibited the lowest PRS, while the ascending-high group displayed the highest PRS. Inconsistent with our results, Riglin et al. reported that PRS scores were highest in the persistent group with ADHD symptoms in children and adolescents [57]. This discrepancy may be attributed to differences in data modeling approaches employed across studies. Riglin et al.’s study utilized binary scores (ADHD or non-ADHD), which limited their ability to capture detailed symptom trajectory information. For instance, their persistence group could only discern transitions from non-ADHD to ADHD without observing subsequent symptom exacerbation after this transition; thus they classified it as a persistent group solely based on this criterion. Conversely, our study employed symptom scores enabling us to observe sustained increases in symptoms over time, leading us to define an ascending-high group. Therefore, the ascending-high group observed in this study and the symptoms-persistent group identified by Riglin et al. are essentially identical. Additionally, our analysis showed that PRS for ADHD ranked second highest in the decreasing-medium group and was lowest in the stable-low group. These provide a theoretical foundation for grouping interventions based on genetic risk in advance.

### Limitations

This study investigated the developmental trajectory of ADHD symptoms in children and adolescents, identifying three distinct trajectories and elucidating the pivotal brain regions underlying the manifestation of ADHD symptoms as well as their genetic basis. However, several limitations exist in this study. First, the longitudinal design only encompassed three time points, which although spanned a period of 3 years, could benefit from more frequent assessments (e.g., two to three times per year) and longer follow-up periods to yield stronger effects. Second, while SDQ was utilized for measuring ADHD symptoms in this study, it should be noted that SDQ is not a comprehensive clinical assessment or validated structured interview; it has good specificity but poor sensitivity [102]. Furthermore, the SDQ did not differentiate between the subdimensions of inattention and hyperactivity. Consequently, it was not possible to distinguish the developmental trajectory of ADHD symptoms along these two subdimensions or explore their respective brain and genetic bases. Additionally, an important point worth considering is that the SDQ does not measure impulsivity. Future studies may consider employing clinical assessment tools or validated structured interview that capture both dimensions of hyperactivity/impulsivity and inattention. Third, given the crucial role played by fronto-striatal circuitry in ADHD pathophysiology, we employed VBM analysis to investigate both cortical and subcortical contributions; however, significant differences were only observed within cortical regions. For the analysis of the cortex, Freesurfer has more advantages, therefore, future analysis of the cortex can use Freesurfer for more accurate segmentation measurement. Finally, we observed a marginal level of significance in the difference between the ascending-high and descending-medium groups, suggesting that caution should be exercised when interpreting these findings in future studies.

## Conclusions

In this study, we employed a LCMM to classify the developmental trajectory of ADHD symptoms in children and adolescents into three distinct groups: ascending-high, stable-low, and descending-medium based on a longitudinal cohort dataset spanning three years. Utilizing the LME model, we identified attention hub regions as the neural basis for these distinct developmental trajectories. These regions encompassed the left ACC/mPFC responsible for inhibitory control, the right IPL involved in directing attention towards external stimuli, and the bilateral MFG/PCG accountable for regulating both dorsal and ventral attention networks while playing a crucial role in flexibly modulating internal and external attentional focus. Through PRS analysis, we have revealed that the ascending-high group of ADHD symptoms across childhood and adolescence in the general population showed the highest PRS for ADHD. This provides a neurobiological and genetic foundation for understanding the distinct developmental trajectory of ADHD symptoms.

### Supplementary Information

Supplementary Material 1.

## Data Availability

The raw data supporting the conclusions of this article were from the Children School Functions and Brain Development Project (CBD, Beijing Cohort), which will be soon made public.

## Abbreviations

ACC/mPFC: Anterior cingulate cortex/medial prefrontal cortex
ADHD: Attention-deficit hyperactivity disorder
BIC: Bayesian information criterion
CAT12: Computational Anatomy Toolbox
CBD: Children School Functions and Brain Development project
DAN: Dorsal attention network
DMN: Default mode network
FDR: False discovery rate
FWE: Family-wise error
GMV: Gray matter volume
IFG: Inferior frontal gyrus
IPL: Inferior parietal lobule
IQR: Image and preprocessing quality
LCMM: Latent class mixed model
LME: Linear mixed-effects
MFG: Middle frontal gyrus
MFG/PCG: Middle frontal gyrus/precentral gyrus
PRS: Polygenic risk scores
SDQ: Strengths and Difficulties Questionnaire
TPJ: Temporo-parietal junction
VAN: Ventral attention network

## References

[1] Thapar A, Cooper M (2016). Attention deficit hyperactivity disorder. Lancet.

[2] Thapar A, Cooper M, Rutter M (2017). Neurodevelopmental disorders. Lancet Psychiat.

[3] American Psychiatric Association D, Association AP. Diagnostic and statistical manual of mental disorders: DSM-5, vol. 5. Washington DC: American Psychiatric Association; 2013.

[4] Faraone SV, Biederman J, Mick E (2006). The age-dependent decline of attention deficit hyperactivity disorder: a meta-analysis of follow-up studies. Psychol Med.

[5] Agnew-Blais JC, Polanczyk GV, Danese A, Wertz J, Moffitt TE, Arseneault L (2016). Evaluation of the persistence, remission, and emergence of attention-deficit/hyperactivity disorder in young adulthood. JAMA Psychiat.

[6] Caye A, Rocha TB, Anselmi L, Murray J, Menezes AM, Barros FC, Goncalves H, Wehrmeister F, Jensen CM, Steinhausen HC (2016). Attention-deficit/hyperactivity disorder trajectories from childhood to young adulthood: evidence from a birth cohort supporting a late-onset syndrome. JAMA Psychiat.

[7] Moffitt TE, Houts R, Asherson P, Belsky DW, Corcoran DL, Hammerle M, Harrington H, Hogan S, Meier MH, Polanczyk GV (2015). Is adult ADHD a childhood-onset neurodevelopmental disorder? Evidence from a four-decade longitudinal cohort study. Am J Psychiat.

[8] Agnew-Blais JC, Polanczyk GV, Danese A, Wertz J, Moffitt TE, Arseneault L (2018). Young adult mental health and functional outcomes among individuals with remitted, persistent and late-onset ADHD. Brit J Psychiat.

[9] Breda V, Rohde LA, Menezes AMB, Anselmi L, Caye A, Rovaris DL, Vitola ES, Bau CHD, Grevet EH (2021). The neurodevelopmental nature of attention-deficit hyperactivity disorder in adults. Brit J Psychiat.

[10] Murray AL, Eisner M, Obsuth I, Ribeaud D (2020). Identifying early markers of "Late Onset" attention deficit and hyperactivity/impulsivity symptoms. J Atten Disord.

[11] Oldham S, Ball G, Fornito A (2022). Early and late development of hub connectivity in the human brain. Curr Opin Psychol.

[12] Griffiths KR, Grieve SM, Kohn MR, Clarke S, Williams LM, Korgaonkar MS (2016). Altered gray matter organization in children and adolescents with ADHD: a structural covariance connectome study. Transl Psychiat.

[13] Wang B, Wang G, Wang X, Cao R, Xiang J, Yan T, Li H, Yoshimura S, Toichi M, Zhao S (2021). Rich-club analysis in adults with ADHD connectomes reveals an abnormal structural core network. J Atten Disord.

[14] Hearne LJ, Lin HY, Sanz-Leon P, Tseng WI, Gau SS, Roberts JA, Cocchi L (2021). ADHD symptoms map onto noise-driven structure-function decoupling between hub and peripheral brain regions. Mol Psychiatr.

[15] Rubia K, Alegría AA, Brinson H (2014). Brain abnormalities in attention-deficit hyperactivity disorder: a review. Rev Neurol.

[16] Casey BJ, Epstein JN, Buhle J, Liston C, Davidson MC, Tonev ST, Spicer J, Niogi S, Millner AJ, Reiss A (2007). Frontostriatal connectivity and its role in cognitive control in parent-child dyads with ADHD. Am J Psychiat.

[17] Cubillo A, Halari R, Smith A, Taylor E, Rubia K (2012). A review of fronto-striatal and fronto-cortical brain abnormalities in children and adults with Attention Deficit Hyperactivity Disorder (ADHD) and new evidence for dysfunction in adults with ADHD during motivation and attention. Cortex.

[18] Frodl T, Skokauskas N (2012). Meta-analysis of structural MRI studies in children and adults with attention deficit hyperactivity disorder indicates treatment effects. Acta Psychiat Scand.

[19] Hart H, Radua J, Nakao T, Mataix-Cols D, Rubia K (2013). Meta-analysis of functional magnetic resonance imaging studies of inhibition and attention in attention-deficit/hyperactivity disorder: exploring task-specific, stimulant medication, and age effects. JAMA Psychiat.

[20] Qiu A, Crocetti D, Adler M, Mahone EM, Denckla MB, Miller MI, Mostofsky SH (2009). Basal ganglia volume and shape in children with attention deficit hyperactivity disorder. Am J Psychiat.

[21] Silk TJ, Vilgis V, Adamson C, Chen J, Smit L, Vance A, Bellgrove MA (2016). Abnormal asymmetry in frontostriatal white matter in children with attention deficit hyperactivity disorder. Brain Imaging Behav.

[22] Valera EM, Faraone SV, Murray KE, Seidman LJ (2007). Meta-analysis of structural imaging findings in attention-deficit/hyperactivity disorder. Biol Psychiat.

[23] Suo XJ, Ding H, Li X, Zhang YD, Liang M, Zhang YQ, Yu CS, Qin W (2021). Anatomical and functional coupling between the dorsal and ventral attention networks. Neuroimage.

[24] Japee S, Holiday K, Satyshur MD, Mukai I, Ungerleider LG (2015). A role of right middle frontal gyrus in reorienting of attention: a case study. Front Syst Neurosci.

[25] Luo Y, Alvarez TL, Halperin JM, Li X (2020). Multimodal neuroimaging-based prediction of adult outcomes in childhood-onset ADHD using ensemble learning techniques. Neuroimage Clin.

[26] Monden Y, Dan H, Nagashima M, Dan I, Tsuzuki D, Kyutoku Y, Gunji Y, Yamagata T, Watanabe E, Momoi MY (2012). Right prefrontal activation as a neuro-functional biomarker for monitoring acute effects of methylphenidate in ADHD children: An fNIRS study. Neuroimage-Clin.

[27] Hoogman M, Bralten J, Hibar DP, Mennes M, Zwiers MP, Schweren LSJ, van Hulzen KJE, Medland SE, Shumskaya E, Jahanshad N (2017). Subcortical brain volume differences in participants with attention deficit hyperactivity disorder in children and adults: a cross-sectional mega-analysis. Lancet Psychiat.

[28] McCarthy H, Skokauskas N, Frodl T (2014). Identifying a consistent pattern of neural function in attention deficit hyperactivity disorder: a meta-analysis. Psychol Med.

[29] Nakao T, Radua J, Rubia K, Mataix-Cols D (2011). Gray matter volume abnormalities in ADHD: voxel-based meta-analysis exploring the effects of age and stimulant medication. Am J Psychiat.

[30] Samea F, Soluki S, Nejati V, Zarei M, Cortese S, Eickhoff SB, Tahmasian M, Eickhoff CR (2019). Brain alterations in children/adolescents with ADHD revisited: a neuroimaging meta-analysis of 96 structural and functional studies. Neurosci Biobehav Rev.

[31] Cortese S, Kelly C, Chabernaud C, Proal E, Di Martino A, Milham MP, Castellanos FX (2012). Toward systems neuroscience of ADHD: a meta-analysis of 55 fMRI studies. Am J Psychiatry.

[32] Dickstein SG, Bannon K, Castellanos FX, Milham MP (2006). The neural correlates of attention deficit hyperactivity disorder: an ALE meta-analysis. J Child Psychol Psyc.

[33] Ellison-Wright I, Ellison-Wright Z, Bullmore E (2008). Structural brain change in attention deficit hyperactivity disorder identified by meta-analysis. BMC Psychiatry.

[34] Norman LJ, Carlisi C, Lukito S, Hart H, Mataix-Cols D, Radua J, Rubia K (2016). Structural and Functional Brain Abnormalities in Attention-Deficit/Hyperactivity Disorder and Obsessive-Compulsive Disorder: a comparative meta-analysis. JAMA Psychiat.

[35] Castellanos FX, Sonuga-Barke EJ, Milham MP, Tannock R (2006). Characterizing cognition in ADHD: beyond executive dysfunction. Trends Cogn Sci.

[36] Rubia K (2011). "Cool" inferior frontostriatal dysfunction in attention-deficit/hyperactivity disorder versus "hot" ventromedial orbitofrontal-limbic dysfunction in conduct disorder: a review. Biol Psychiat.

[37] Ariyarathne G, De Silva S, Dayarathna S, Meedeniya D, Jayarathne S. ADHD identification using convolutional neural network with seed-based approach for fMRI data [Conference session]. In: proceedings of the 2020 9th international conference on software and computer applications, Langkawi, Malaysia; 2020. p. 31–35.

[38] Kuang DP, Guo XJ, An X, Zhao YL, He LH (2014). Discrimination of ADHD Based on fMRI data with deep belief network. Lect N Bioinformat.

[39] Metin B, Krebs RM, Wiersema JR, Verguts T, Gasthuys R, van der Meere JJ, Achten E, Roeyers H, Sonuga-Barke E (2015). Dysfunctional modulation of default mode network activity in attention-deficit/hyperactivity disorder. J Abnorm Psychol.

[40] Sonuga-Barke EJ, Castellanos FX (2007). Spontaneous attentional fluctuations in impaired states and pathological conditions: a neurobiological hypothesis. Neurosci Biobehav Rev.

[41] Norman LJ, Sudre G, Price J, Shastri GG, Shaw P (2023). Evidence from "big data" for the default-mode hypothesis of ADHD: a mega-analysis of multiple large samples. Neuropsychopharmacol.

[42] Castellanos FX, Sonuga-Barke EJ, Scheres A, Di Martino A, Hyde C, Walters JR (2005). Varieties of attention-deficit/hyperactivity disorder-related intra-individual variability. Biol Psychiat.

[43] Dillo W, Goke A, Prox-Vagedes V, Szycik GR, Roy M, Donnerstag F, Emrich HM, Ohlmeier MD (2010). Neuronal correlates of ADHD in adults with evidence for compensation strategies--a functional MRI study with a Go/No-Go paradigm. Ger Med Sci.

[44] Karch S, Thalmeier T, Lutz J, Cerovecki A, Opgen-Rhein M, Hock B, Leicht G, Hennig-Fast K, Meindl T, Riedel M (2010). Neural correlates (ERP/fMRI) of voluntary selection in adult ADHD patients. Eur Arch Psychiatry Clin Neurosci.

[45] Ahrendts J, Rusch N, Wilke M, Philipsen A, Eickhoff SB, Glauche V, Perlov E, Ebert D, Hennig J, van Elst LT (2011). Visual cortex abnormalities in adults with ADHD: a structural MRI study. World J Biol Psychia.

[46] Mostofsky SH, Rimrodt SL, Schafer JG, Boyce A, Goldberg MC, Pekar JJ, Denckla MB (2006). Atypical motor and sensory cortex activation in attention-deficit/hyperactivity disorder: a functional magnetic resonance imaging study of simple sequential finger tapping. Biol Psychiat.

[47] Faraone SV, Perlis RH, Doyle AE, Smoller JW, Goralnick JJ, Holmgren MA, Sklar P (2005). Molecular genetics of attention-deficit/hyperactivity disorder. Biol Psychiat.

[48] Stergiakouli E, Thapar A (2010). Fitting the pieces together: current research on the genetic basis of attention-deficit/hyperactivity disorder (ADHD). Neuropsychiatr Dis Treat.

[49] Pingault JB, Viding E, Galera C, Greven CU, Zheng Y, Plomin R, Rijsdijk F (2015). Genetic and environmental influences on the developmental course of attention-deficit/hyperactivity disorder symptoms from childhood to adolescence. JAMA Psychiat.

[50] Chang Z, Lichtenstein P, Asherson PJ, Larsson H (2013). Developmental twin study of attention problems: high heritabilities throughout development. JAMA Psychiat.

[51] Franke B, Faraone SV, Asherson P, Buitelaar J, Bau CH, Ramos-Quiroga JA, Mick E, Grevet EH, Johansson S, Haavik J (2012). The genetics of attention deficit/hyperactivity disorder in adults, a review. Mol Psychiatr.

[52] Larsson H, Asherson P, Chang Z, Ljung T, Friedrichs B, Larsson JO, Lichtenstein P (2013). Genetic and environmental influences on adult attention deficit hyperactivity disorder symptoms: a large Swedish population-based study of twins. Psychol Med.

[53] Thapar A, Cooper M, Eyre O, Langley K (2013). What have we learnt about the causes of ADHD?. J Child Psychol Psyc.

[54] Hamshere ML, Langley K, Martin J, Agha SS, Stergiakouli E, Anney RJ, Buitelaar J, Faraone SV, Lesch KP, Neale BM (2013). High loading of polygenic risk for ADHD in children with comorbid aggression. Am J Psychiatry.

[55] Martin J, Hamshere ML, Stergiakouli E, O'Donovan MC, Thapar A (2014). Genetic risk for Attention-Deficit/Hyperactivity Disorder contributes to neurodevelopmental traits in the general population. Mol Psychiatr.

[56] Groen-Blokhuis MM, Middeldorp CM, Kan KJ, Abdellaoui A, van Beijsterveldt CE, Ehli EA, Davies GE, Scheet PA, Xiao X, Hudziak JJ (2014). Attention-Deficit/Hyperactivity Disorder polygenic risk scores predict attention problems in a population-based sample of children. J Am Acad Child Adolesc Psychiatry.

[57] Riglin L, Collishaw S, Thapar AK, Dalsgaard S, Langley K, Smith GD, Stergiakouli E, Maughan B, O'Donovan MC, Thapar A (2016). Association of genetic risk variants with Attention-Deficit/Hyperactivity Disorder trajectories in the general population. JAMA Psychiat.

[58] Tao S (2019). Intelligence development and school adjustment of school-age children and adolescents: a follow-up cohort study. Psychol Commun.

[59] Dong Q, Lin CD (2011). Standardized tests in children and adolescent mental development in China.

[60] Sheehan DV, Lecrubier Y, Sheehan KH, Amorim P, Janavs J, Weiller E, Hergueta T, Baker R, Dunbar GC (1998). The Mini-International Neuropsychiatric Interview (M.I.N.I.): the development and validation of a structured diagnostic psychiatric interview for DSM-IV and ICD-10. J Clin Psychiat.

[61] Goodman R (1997). The strengths and difficulties questionnaire: a research note. J Child Psychol Psyc.

[62] Albaugh MD, Hudziak JJ, Ing A, Chaarani B, Barker E, Jia T, Lemaitre H, Watts R, Orr C, Spechler PA (2019). White matter microstructure is associated with hyperactive/inattentive symptomatology and polygenic risk for attention-deficit/hyperactivity disorder in a population-based sample of adolescents. Neuropsychopharmacol.

[63] Albaugh MD, Ivanova M, Chaarani B, Orr C, Allgaier N, Althoff RR, D’Alberto N, Hudson K, Mackey S, Spechler PA (2019). Ventromedial prefrontal volume in adolescence predicts hyperactive/inattentive symptoms in adulthood. Cereb Cortex.

[64] Albaugh MD, Orr C, Chaarani B, Althoff RR, Allgaier N, D'Alberto N, Hudson K, Mackey S, Spechler PA, Banaschewski T (2017). Inattention and reaction time variability are linked to ventromedial prefrontal volume in adolescents. Biol Psychiat.

[65] Bayard F, Nymberg Thunell C, Abe C, Almeida R, Banaschewski T, Barker G, Bokde ALW, Bromberg U, Buchel C, Quinlan EB (2020). Distinct brain structure and behavior related to ADHD and conduct disorder traits. Mol Psychiatr.

[66] Shrout PE (1998). Measurement reliability and agreement in psychiatry. Stat Methods Med Res.

[67] Goodman R (2001). Psychometric properties of the strengths and difficulties questionnaire. J Am Acad Child Adolesc Psychiatry.

[68] Wang Y, Guan H, Ma L, Luo J, Chu C, Hu M, Zhao G, Men W, Tan S, Gao JH (2022). Learning to read may help promote attention by increasing the volume of the left middle frontal gyrus and enhancing its connectivity to the ventral attention network. Cereb Cortex.

[69] Marcus DS, Harms MP, Snyder AZ, Jenkinson M, Wilson JA, Glasser MF, Barch DM, Archie KA, Burgess GC, Ramaratnam M (2013). Human connectome project informatics: quality control, database services, and data visualization. Neuroimage.

[70] Murray AL, Hall HA, Speyer LG, Carter L, Mirman D, Caye A, Rohde L (2022). Developmental trajectories of ADHD symptoms in a large population-representative longitudinal study. Psychol Med.

[71] Proust-Lima C, Philipps V, Liquet B (2017). Estimation of extended mixed models using latent classes and latent processes: The R package lcmm. J Stat Softw.

[72] Yan CG, Wang XD, Zuo XN, Zang YF (2016). DPABI: data processing & analysis for (Resting-State) brain imaging. Neuroinformatics.

[73] Bernal-Rusiel JL, Greve DN, Reuter M, Fischl B, Sabuncu MR (2013). Alzheimer’s Disease Neuroimaging I: statistical analysis of longitudinal neuroimage data with Linear Mixed Effects models. Neuroimage.

[74] Eklund A, Nichols TE, Knutsson H (2016). Cluster failure: why fMRI inferences for spatial extent have inflated false-positive rates. Proc Natl Acad Sci U S A.

[75] Dennis M, Francis DJ, Cirino PT, Schachar R, Barnes MA, Fletcher JM (2009). Why IQ is not a covariate in cognitive studies of neurodevelopmental disorders. J Int Neuropsychol Soc.

[76] Demontis D, Walters GB, Athanasiadis G, Walters R, Therrien K, Nielsen TT, Farajzadeh L, Voloudakis G, Bendl J, Zeng B (2023). Genome-wide analyses of ADHD identify 27 risk loci, refine the genetic architecture and implicate several cognitive domains. Nat Genet.

[77] Choi SW, O'Reilly PF. PRSice-2: polygenic risk score software for biobank-scale data. Gigasci. 2019;8(7):giz082. 10.1093/gigascience/giz082 PMC6629542 31307061

[78] Benjamini Y, Drai D, Elmer G, Kafkafi N, Golani I (2001). Controlling the false discovery rate in behavior genetics research. Behav Brain Res.

[79] Olsson-Collentine A, van Assen M, Hartgerink CHJ (2019). The prevalence of marginally significant results in psychology over time. Psychol Sci.

[80] Waller R, Hyde LW, Grabell AS, Alves ML, Olson SL (2015). Differential associations of early callous-unemotional, oppositional, and ADHD behaviors: multiple domains within early-starting conduct problems?. J Child Psychol Psyc.

[81] Forslund T, Brocki KC, Bohlin G, Granqvist P, Eninger L (2016). The heterogeneity of attention-deficit/hyperactivity disorder symptoms and conduct problems: cognitive inhibition, emotion regulation, emotionality, and disorganized attachment. Brit J Dev Psychol.

[82] Volotinen L, Metsä-Simola N, Remes H, Martikainen P. Parental education, ADHD in family and ADHD diagnosis incidence in Finnish children and adolescents. Eur J Public Health. 2023;33.Supplement_2(2023):ckad160–542

[83] Russell AE, Ford T, Williams R, Russell G (2016). The association between socioeconomic disadvantage and Attention Deficit/Hyperactivity Disorder (ADHD): a systematic review. Child Psychiatry Hum Dev.

[84] Riglin L, Wootton RE, Livingston LA, Agnew-Blais J, Arseneault L, Blakey R, Agha SS, Langley K, Collishaw S, O'Donovan MC (2022). “Late-onset” ADHD symptoms in young adulthood: is this ADHD?. J Atten Disord.

[85] Ek U, Fernell E, Westerlund J, Holmberg K, Olsson PO, Gillberg C (2007). Cognitive strengths and deficits in schoolchildren with ADHD. Acta Paediatr.

[86] Kuntsi J, Eley TC, Taylor A, Hughes C, Asherson P, Caspi A, Moffitt TE (2004). Co-occurrence of ADHD and low IQ has genetic origins. Am J Med Genet B.

[87] Agnew-Blais JC, Polanczyk GV, Danese A, Wertz J, Moffitt TE, Arseneault L (2020). Are changes in ADHD course reflected in differences in IQ and executive functioning from childhood to young adulthood?. Psychol Med.

[88] Huttenlocher PR, Dabholkar AS (1997). Regional differences in synaptogenesis in human cerebral cortex. J Comp Neurol.

[89] Sowell ER, Thompson PM, Toga AW (2004). Mapping changes in the human cortex throughout the span of life. Neuroscientist.

[90] Makris N, Seidman LJ, Valera EM, Biederman J, Monuteaux MC, Kennedy DN, Caviness VS, Bush G, Crum K, Brown AB (2010). Anterior cingulate volumetric alterations in treatment-naive adults with ADHD: a pilot study. J Atten Disord.

[91] Castellanos FX, Margulies DS, Kelly C, Uddin LQ, Ghaffari M, Kirsch A, Shaw D, Shehzad Z, Di Martino A, Biswal B (2008). Cingulate-precuneus interactions: a new locus of dysfunction in adult attention-deficit/hyperactivity disorder. Biol Psychiat.

[92] Fassbender C, Zhang H, Buzy WM, Cortes CR, Mizuiri D, Beckett L, Schweitzer JB (2009). A lack of default network suppression is linked to increased distractibility in ADHD. Brain Res.

[93] Fox MD, Corbetta M, Snyder AZ, Vincent JL, Raichle ME (2006). Spontaneous neuronal activity distinguishes human dorsal and ventral attention systems. Proc Natl Acad Sci U S A.

[94] Corbetta M, Patel G, Shulman GL (2008). The reorienting system of the human brain: from environment to theory of mind. Neuron.

[95] Wen X, Yao L, Liu Y, Ding M (2012). Causal interactions in attention networks predict behavioral performance. J Neurosci.

[96] Chen H, Liu K, Zhang B, Zhang J, Xue X, Lin Y, Zou D, Chen M, Kong Y, Wen G (2019). More optimal but less regulated dorsal and ventral visual networks in patients with major depressive disorder. J Psychiatr Res.

[97] Shapiro K, Hillstrom AP, Husain M (2002). Control of visuotemporal attention by inferior parietal and superior temporal cortex. Curr Biol.

[98] Wilterson AI, Nastase SA, Bio BJ, Guterstam A, Graziano MSA. Attention, awareness, and the right temporoparietal junction. Proc Natl Acad Sci USA. 2021;118(25):e2026099118. 10.1073/pnas.2026099118 PMC8237657 34161276

[99] Seitz J, Hueck M, Dahmen B, Schulte-Ruther M, Legenbauer T, Herpertz-Dahlmann B, Konrad K (2016). Attention network dysfunction in bulimia nervosa - an fMRI Study. PLoS One.

[100] Helenius P, Laasonen M, Hokkanen L, Paetau R, Niemivirta M (2011). Impaired engagement of the ventral attentional pathway in ADHD. Neuropsychologia.

[101] Fusar-Poli P, Rubia K, Rossi G, Sartori G, Balottin U (2012). Striatal dopamine transporter alterations in ADHD: pathophysiology or adaptation to psychostimulants? A meta-analysis. Am J Psychiat.

[102] Hall CL, Guo B, Valentine AZ, Groom MJ, Daley D, Sayal K, Hollis C (2019). The validity of the strengths and difficulties questionnaire (SDQ) for children with ADHD symptoms. PLoS One.

