# Supplementary material for: The neural and genetic underpinnings of different developmental trajectories of Attention-Deficit/Hyperactivity Symptoms in children and adolescents
Source: BMC Med. 2024 Jun 3;22:223. doi: 10.1186/s12916-024-03449-1 (PMC11149188; doi:10.1186/s12916-024-03449-1)
Supplement: Supplementary file 1 — Supplementary Material 1. [file 12916_2024_3449_MOESM1_ESM.docx]

Supplementary Information

**The neural and genetic underpinnings of different developmental trajectories of Attention-Deficit/Hyperactivity Symptoms in children and adolescents**

Yanpei Wang, et al.

**Table of Content**

[Supplementary methods 2](#_Toc166837121)

[Method S1. MRI Acquisition, quality controls and Image Processing 2](#_Toc166837122)

[Method S2. Genotyping 3](#_Toc166837123)

[Method S3. The syntax of latent class mixed model and linear mixed-effects model 3](#_Toc166837124)

[Method S4. Demographic Measures 4](#_Toc166837125)

[Supplementary tables 5](#_Toc166837126)

[Table S1. The fitting solution from one class to seven class 5](#_Toc166837127)

[Table S2. The age effect on GMV 5](#_Toc166837128)

[Table S3. The group (descending, stable and ascending) effect on GMV 6](#_Toc166837129)

[Table S4. The interaction of age and group on GMV 6](#_Toc166837130)

[Supplementary figures 7](#_Toc166837131)

[Figure S1. The different developmental trajectories of emotional symptoms and conduct problems 7](#_Toc166837132)

# Supplementary methods

## Method S1. MRI Acquisition, quality controls and Image Processing

All MRI scans were acquired on the same 3T Siemens Prisma 64-channel head coil at Beijing Normal University, Peking University and Beijing HuiLongGuan Hospital using the same imaging sequences. A 6-minute magnetization-prepared, rapid acquisition gradient-echo T1-weighted (MPRAGE) image (TR=2530 ms, TE 2.98 ms, FOV 256 mm ×224 mm, matrix, effective voxel resolution of 0.5×0.5×1 mm^3^, slice thickness =1 mm, and slices=192) was acquired to aid spatial normalization to standard atlas space. Prior to scanning, to acclimate subjects (children) to the MRI environment, a mock scanning session was conducted for each individual using a decommissioned MRI scanner and head coil. Mock scanning was accompanied by acoustic recordings of the noise produced by gradient coils for each scanning pulse sequence. To further minimize motion, subjects’ heads were stabilized in the head coil using one foam pad over each ear.

All MRI scan quality control procedures are described below. i) Individual images were subjected to a careful visual examination by an experienced radiologist to exclude incidental abnormalities, such as arachnoid cysts, neuroepithelial cysts and other intracranial space-occupying lesions. ii) Careful visual inspections with a scan rating procedure were separately conducted by five experienced raters using a protocol similar to that used in the Human Connectome Project. iii) Images considered to have a better than fair quality by both raters were retained.

The voxel-based morphometry (VBM) was conducted using the Computational Anatomy Toolbox (CAT12) (http://dbm.neuro.uni-jena.de/cat) and SPM12 (http://www.fil.ion.ucl.ac.uk/spm). T1 images were segmented into gray matter, white matter, and cerebrospinal fluid based on the default settings of CAT12. Next, images were normalized to Montreal Neurological Institute space using the DARTEL approach. The registered gray matter images were multiplied with the Jacobian determinants derived from the spatial normalization (voxel size 2 mm^3^) process and then smoothed with a 6-mm full-width at half-maximum Gaussian kernel.

## Method S2. Genotyping

DNA purification and genotyping were performed by the CapitalBio Technology, Beijing. DNA was extracted from whole-blood samples (∼6 mL) preserved in BD Vacutainer EDTA Tubes (Becton, Dickinson and Company) according to the manufacturer’s instructions. After preliminary quality control, genotype imputation was conducted using IMPUTE2 and referencing the genetic sequence of the East Asian population in 1000 Genome Phase3. SNPs with call rates of <99%, minor allele frequency <1%, or deviation from the Hardy–Weinberg equilibrium (*p*<1.00×10^−6^) were excluded from the analyses. Individuals with an ambiguous sex code, close genetic relationship (rel-cutoff=0.125), excessive missing genotypes (failure rate>1%), and outlying heterozygosity (heterozygosity rate of 3 SDs from the mean) were also excluded.

## Method S3. The syntax of latent class mixed model and linear mixed-effects model

We used a latent class mixed model (LCMM) on ADHD symptoms data to ascertain different trajectories of ADHD symptoms from 487 individuals of the CBD cohort. The syntax of LCMM is as follows:

hlme(fixed, mixture, random, subject, classmb, ng = 1, idiag = FALSE, nwg = FALSE, cor = NULL, data, B, convB = 0.0001, convL = 0.0001, convG = 0.0001, prior, maxiter = 500, subset = NULL, na.action = 1, posfix = NULL)

Then, we used a linear mixed model to quantify the effects of groups of ADHD symptoms and age on the gray matter volume. The syntax of linear mixed-effects model is as follows:

lme(fixed, data, random, correlation, weights, subset, method, na.action, control, contrasts = NULL, keep.data = TRUE)

## Method S4. Demographic Measures

The parent's education level refers to the mean level of education between children’s parents. Parental Education: 1 = Uneducated; 2 = Primary education; 3 = Junior school; 4 = High school or Secondary vocational school or Polytechnic school; 5 = Higher vocational education or Junior college(part-time) or Junior college(full-time); 6 = Bachelor degree (part-time); 7 = Bachelor degree (full-time); 8 = Graduate education or above. Family Income (RMB/year): 1 = Less than 3000; 2 = 3001-6000; 3 = 6001-10000; 4 = 10001-30000; 5 = 30001-50000; 6 = 50001-100000; 7 = 100001-150000; 8 = 150001-200000; 9 = 200001-400000; 10 = 400001-600000; 11 = Over 600000.

# Supplementary tables

## Table S1. The fitting solution from one class to seven class

| Group | BIC | Class1 | Class2 | Class3 | Class4 | Class5 | Class6 | Class7 |
| --- | --- | --- | --- | --- | --- | --- | --- | --- |
| 1 | 6031.02 | 100% |  |  |  |  |  |  |
| 2 | 6027.43 | 6.98% | 93.02% |  |  |  |  |  |
| **3** | **6018.98** | **79.06%** | **7.60%** | **13.35%** |  |  |  |  |
| 4 | 6042.91 | 6.98% | 14.37% | 12.11% | 66.53% |  |  |  |
| 5 | 6061.11 | 5.54% | 68.79% | 4.11% | 14.37% | 7.19% |  |  |
| 6 | 6085.39 | 5.54% | 14.37% | 49.08% | 5.54% | 19.92% | 5.54% |  |
| 7 | 6107.78 | 7.19% | 4.31% | 42.09% | 6.16% | 24.85% | 15.40% | 0 |

## Table S2. The age effect on GMV

| Anatomical Regions | HS | MNI Coordinate | | | Voxel | *z*-value |
| --- | --- | --- | --- | --- | --- | --- |
|  |  | x | y | z |  |  |
| Precuneus / Sub-lobar / Sub-Gyral / Middle Occipital Gyrus / Cuneus / Middle Temporal Gyrus / Cingulate Gyrus / Middle Frontal Gyrus / Lentiform Nucleus / Superior Temporal Gyrus Extra-Nuclear / Superior Frontal Gyrus Lingual Gyrus / Putamen / Posterior Cingulate / Superior Parietal Lobule / Inferior Frontal Gyrus / Inferior Parietal Lobule Postcentral Gyrus / Paracentral Lobule / Inferior Occipital Gyrus / Thalamus Insula / Inferior Temporal Gyrus / Angular Gyrus Ventral Lateral Nucleus / Medial Frontal Gyrus / Medial Globus Pallidus / Supramarginal Gyrus / Fusiform Gyrus | L | -21 | -57 | -18 | 32461 | -9.04 |
| Middle Frontal Gyrus / Superior Frontal Gyrus / Precentral Gyrus | L | -15 | -3 | 66 | 1739 | 6.80 |
| Superior Frontal Gyrus / Middle Frontal Gyrus | R | 15 | 24 | 54 | 466 | 5.51 |
| Inferior Frontal Gyrus / Sub-Gyral / Sub-lobar / Extra-Nuclear / Insula | R | 18 | 39 | -12 | 462 | -6.77 |
| Fusiform Gyrus / Inferior Temporal Gyrus / Middle Temporal Gyrus | R | 27 | -6 | -45 | 355 | 5.10 |
| Caudate / Anterior Cingulate | L | -6 | 18 | 3 | 278 | -5.59 |
| Middle Frontal Gyrus / Superior Frontal Gyrus | L | -24 | 48 | 6 | 215 | -6.59 |
| Middle Frontal Gyrus | R | 30 | -3 | 51 | 196 | 4.67 |

*Abbreviation*: HS = hemisphere; L = left; R = right; GMV = gray matter volume.

## Table S3. The group (descending, stable and ascending) effect on GMV

| Anatomical Regions | HS | MNI Coordinate | | | Voxel | *z*-value |
| --- | --- | --- | --- | --- | --- | --- |
|  |  | x | y | z |  |  |
| Inferior Parietal Lobule / Supramarginal Gyrus | R | 33 | -42 | 39 | 237 | 5.17 |

*Abbreviation*: HS = hemisphere; L = left; R = right; GMV = gray matter volume.

## Table S4. The interaction of age and group on GMV

| Anatomical Regions | HS | MNI Coordinate | | | Voxel | *z*-value |
| --- | --- | --- | --- | --- | --- | --- |
|  |  | x | y | z |  |  |
| Anterior Cingulate / Medial Frontal Cortex | L | -15 | 48 | 0 | 485 | -4.77 |
| Inferior Parietal Lobule / Supramarginal Gyrus | R | 30 | -42 | 39 | 338 | -5.20 |
| Precentral Gyrus / Middle Frontal Gyrus | R | 33 | -21 | 42 | 362 | -5.11 |
| Precentral Gyrus / Middle Frontal Gyrus | L | -24 | -21 | 66 | 255 | -4.96 |

*Abbreviation*: HS = hemisphere; L = left; R = right; GMV = gray matter volume.

# Supplementary figures

## Figure S1. The different developmental trajectories of emotional symptoms and conduct problems

**
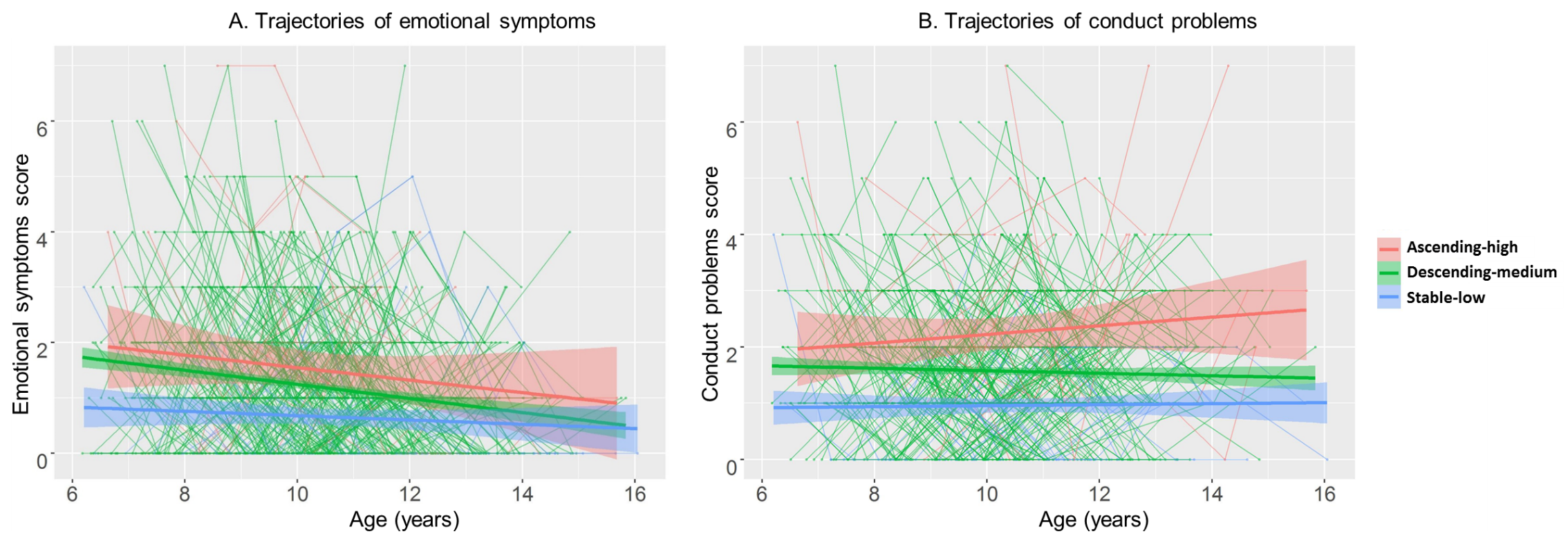
**

Figure S1. The different developmental trajectories of emotional symptoms and conduct problems. A. The different developmental trajectories of emotional symptoms; B. The different developmental trajectories of conduct problems. The shaded areas represent the 95% confidence intervals. Individual participants are represented by individual lines, and participants measured once are represented by dots.
